# Birth weight for gestational age and later cardiovascular health: a comparison between longitudinal Finnish and indigenous Australian cohorts

**DOI:** 10.1080/07853890.2021.1999491

**Published:** 2021-11-10

**Authors:** Pauline Sjöholm, Katja Pahkala, Belinda Davison, Harri Niinikoski, Olli Raitakari, Markus Juonala, Gurmeet R. Singh

**Affiliations:** a Department of Medicine; University of Turku, Turku, Finland; b Division of Perioperative Services, Intensive Care and Pain Medicine, Turku University Hospital, Turku, Finland; c Research Centre of Applied and Preventive Cardiovascular Medicine, University of Turku, Turku, Finland; d Centre for Population Health Research, University of Turku and Turku University Hospital, Finland; e Paavo Nurmi Centre, Sports & Exercise Medicine Unit, Department of Physical Activity and Health, University of Turku, Turku, Finland; f Menzies School of Health Research, Charles Darwin University, Darwin, Australia; g Department of Pediatrics, Turku University Hospital, Finland; h Department Clinical Physiology and Nuclear Medicine, Turku University Hospital, Finland; i Division of Medicine, Turku University Hospital, Turku, Finland; j Northern Territory Medical Program, Flinders University, Darwin, Australia

**Keywords:** Birth weight, gestational age, foetal growth, cardiovascular, risk factors, longitudinal study, aboriginal, indigenous

## Abstract

**Introduction:**

Small or large birth weight for gestational age has been linked with later cardiovascular disease risk. However, cardiovascular risk markers from childhood to adulthood according to birth weight in diverse longitudinal settings globally have not been extensively studied.

**Objectives:**

To examine the relationship between birth weight and cardiovascular risk profile from childhood until young adulthood in two geographically and socioeconomically distinct cohorts.

**Methods:**

Data were derived from two longitudinal birth cohort studies; one from southern Finland (Special Turku Coronary Risk Factor Intervention Project, STRIP) and one from northern Australia comprising Indigenous Australians (Aboriginal Birth Cohort, ABC). The sample included 747 Finnish participants and 541 Indigenous Australians with data on birth weight, gestational age and cardiovascular risk factors (body mass index [BMI]), waist-to-height ratio [WHtR], lipid profile, blood pressure) collected at ages 11, 18 and 25 or 26 years. Carotid intima-media thickness (cIMT) was assessed at age 18 or 19 years. Participants were categorised according to birth weight for gestational age (small [SGA], appropriate [AGA] or large [LGA]). Associations between birth weight category and cardiovascular risk markers were studied using a repeated measures ANOVA.

**Results:**

Higher birth weight category was associated with higher BMI later in life in both cohorts (*p*=.003 for STRIP and *p*<.0001 for ABC). In the ABC, higher birth weight category was also associated with higher WHtR (*p*=.004). In the ABC, SGA participants had lower systolic and diastolic blood pressure than AGA participants (*p*=.028 for systolic, *p*=.027 for diastolic) and lower systolic blood pressure than LGA participants (*p*=.046) at age 25. In the STRIP cohort, SGA participants had lower cIMT than LGA participants (*p*=.024).

**Conclusions:**

Birth weight can predict future cardiovascular risk profile in diverse populations. Thus, it needs to be included in targeted public health interventions for tackling the obesity pandemic and improving cardiovascular health worldwide.

**Key messages**
- The strongest association between birth weight and later cardiovascular risk profile was manifested as differences in body mass index in two culturally and geographically distinct cohorts.
- Foetal growth is a determinant for later cardiovascular health in diverse populations, indicating a need to focus on maternal and foetal health to improve cardiovascular health worldwide.

## Introduction

The British epidemiologist David Barker proposed in 1990 that foetal undernutrition leading to disproportionate foetal growth, predisposes to coronary heart disease later in life [1]. This hypothesis and the resulting approach called the Developmental Origins of Health and Disease (DOHaD) has yielded vast amounts of literature examining the relationship between poor foetal growth, childhood circumstances and the resulting health or disease trajectories during an individual’s life course [2,3]. Foetal programming that takes place in an adverse intrauterine environment may manifest as either a lower or higher birth weight than expected, and can predispose an individual to a variety of chronic diseases, including disorders of the cardiovascular, respiratory, musculoskeletal and neural organ systems [4].

Cardiovascular disease is the leading cause of death worldwide [5]. Individuals’ genetic and epigenetic properties, and their interactions with the environment are thought to drive the processes that lead to the accumulation of cardiovascular risk factors and the subsequent development of cardiovascular disease [6]. Recent studies suggest that the association between birth weight and cardiovascular risk is *J*- or *U*-shaped rather than inverse, indicating that both low and high birth weight are risk factors for cardiovascular disease [6,7]. The association between low birth weight and later CVD risk has been shown in several populations, including specific cohorts such as Indigenous Australians [8], but it remains unclear, whether low and high birthweight affect later cardiovascular risk profiles in a similar manner across different cultural and geographical settings [9].

In this study we examined links between birth weight status and cardiovascular health markers including arterial intima-media thickness at ages 11, 18 and 25 to 26 in two prospective birth cohorts from opposite sides of the globe, Finland and Australia. The two cohorts are nearly contemporaneous but differ considerably in terms of geography, culture, and socioeconomic circumstances, thus providing a unique research setting. Our hypothesis was that disproportionate foetal growth predisposes to poor cardiovascular health irrespective of geographical or cultural setting.

## Methods

The Finnish cohort comes from an urban area with nutritional security and high levels of education [10]. In contrast, the Australian cohort comprises Indigenous individuals from the largely rural and remote Northern Australia with high rates of communicable diseases, unemployment, food insecurity and overcrowded housing [11]. By applying data from these two unique cohorts, the aim of this study was to investigate the associations between birth weight and later cardiovascular health indicated by body mass index (BMI), waist-to-height ratio (WHtR), blood pressure (systolic, diastolic and pulse pressure), serum lipid levels (total cholesterol, high-density lipoprotein [HDL] and low-density lipoprotein [LDL] cholesterol, and triglycerides) as well as carotid intima-media thickness (cIMT).

## Participants

### The aboriginal birth cohort (ABC), Northern Territory, Australia

The ABC study was created to investigate the possible developmental origins behind the high rates of non-communicable disease in the Aboriginal population in Australia. It is one of the longest running and largest indigenous birth cohorts in Australasia. Between 1987 and 1990, 686 of the 1,238 singleton babies born to Indigenous mothers at the Royal Darwin Hospital were recruited into the study. There were no significant differences in mean birth weight and sex ratio between those recruited and those not recruited. The majority of the participants (75%) resided in remote communities across the sparsely populated northern Australia and 25% lived in urban Darwin or its immediate surroundings at the time of recruitment, comparable to the Northern Australian Indigenous population [12]. There have been three follow-ups (waves) to date: Wave-2 in 1998–2001 (mean age 11.4 years), Wave-3 in 2006–2008 (mean age 18.2 years) and Wave-4 in 2014–2016 (mean age 25.4 years) [11,13].

### The special Turku coronary risk factor intervention project (STRIP), Turku, Finland

The STRIP study is a continuing dietary intervention trial that was launched in 1989 in Turku, an urban area in southwest Finland. The aim of the intervention was to promote cardiovascular health by introducing a heart-healthy diet beginning from infancy. Recruitment of the children and their parents was done at well-baby clinics by nurses during a 5-month visit. Between February 1990 and June 1992, 1,062 babies were enrolled and equally allocated to an intervention or control group. Thereafter, the intervention was continued for 20 years, and extensive data focussing on diet and cardiovascular health were obtained at least annually to the age of 20 years [10]. After the intervention period, the first follow-up of the cohort was completed at age 26 years (*n* = 551) [14].

In this study, the STRIP intervention and control groups were treated combined as there was no difference in birth weight (*p*=.20) or birth weight category (*p*=.83) between the groups.

The participants in this study included those for whom data on birth weight, gestational age and at least one cardiovascular marker in the follow-ups were available. Very preterm infants (gestational age less than 33 weeks) and infants with extremely low birth weight (less than 1,000 grams) were excluded. In total, 541 infants from the ABC and 747 infants from the STRIP study were included in the analyses.

## Ethics approval

This study was approved by the Human Research Ethics Committee of the Northern Territory Department of Health and Menzies School of Health Research, including the Aboriginal Ethics Sub-committee with the power of veto (reference, 2013–2022). The investigation complied with the National Health and Medical Research Council National Statement on Ethical Conduct in Human Research (2007) and the Helsinki Declaration of 1975 (2008 revision). Informed written consent was obtained from all participants or (until the age of 18 years) their mothers. The STRIP study was approved by the associated university and hospital district ethical authorities. Written informed consent was obtained from parents at study entry, and from the participants at ages 15, 18, and 26 years.

## Procedures

### Birth weight and gestational age

In the ABC, infant birth weight was measured within 2 h of delivery. Birth weights were recorded to the nearest gram using a balance scale. A neonatal paediatrician performed a gestational age assessment on the participants according to the Dubowitz scoring system [15] within 4 days of birth. In the STRIP study, data on gestational age and birth weight were collected from records of well-baby clinics.

Using an international growth reference Intergrowth-21st, the participants from both cohorts were classified as small for gestational age (SGA; <10th percentile of birthweight for gestational age), appropriate for gestational age (AGA; 10–90th percentile for gestational age), or large for gestational age (LGA; >90th percentile of birthweight for gestational age) according to sex [16].

### Cardiovascular markers

In the ABC, venous blood samples were collected for assessing serum lipid levels (total, LDL- and HDL-cholesterol, and triglycerides) by enzymatic methods (analytic devices employed: Hitachi 917, Roche, XPand Plus, Siemens). Blood pressure was measured three times during each follow-up (right arm, sitting after resting) with an automatic oscillatory unit (LifeSign 420, Welch Allyn); the mean systolic and diastolic values were included in the analyses. Weight was measured to 0.1 kg with a digital scale (TBF-521, Tanita) while the participant was barefoot and in light clothing. Height was measured with a portable stadiometer to the nearest millimetre. Waist circumference was measured to the nearest millimetre using a flexible tape measure at the midpoint between the lowest rib and iliac crest at the end of exhalation [13,17]. CIMT at age 18 years was measured using external B-mode ultrasound with the patient lying in a supine position with a SonoHeart Elite System (Sonosite Incorporated, Bothell, WA, USA) and a 10.5 MHz linear array transducer. Two readings from both common carotid arteries were averaged to calculate mean IMT. Readings were taken at end-diastole (on R wave of simultaneously recorded 3-lead ECG tracing) [18].

In the STRIP study, established clinical laboratory methods were used to measure serum lipid levels (total and HDL-cholesterol, and triglycerides) from venous blood samples taken after an overnight fast [19]. LDL-cholesterol concentration was calculated according to the Friedewald formula [20]. Sitting blood pressure was measured two to four times at each visit using an oscillometric device observing appropriate rest time (15 min) and cuff sizes [21]. In the 26-year follow-up, three blood pressure measurements were taken and the mean value was used for analyses [14]. Weight was measured to the nearest 0.1 kg with an electronic scale (S10, Soehnle, Murrhardt, Germany) and height to the nearest millimetre with a stadiometer [22]. Waist circumference was measured midway between the iliac crest and the lowest rib at the midaxillary line to the nearest 0.5 cm with a flexible measuring tape [23]. Ultrasonography was used to assess cIMT (Acuson Sequoia 512 mainframe; Acuson, Mountain View, CA). At age 19, the far wall of the distal common carotid arteries on both sides 1 to 2 cm from the bulb were scanned from anterior oblique and lateral angles using a 13 MHz linear-array transducer [24]. Two end-diastolic frames from both interrogation angles on both sides were analysed. Four measures were obtained in each image; the mean indicated average carotid IMT.

For both cohorts, BMI was calculated as weight (kg) divided by the square of height (m^2^), and WHtR was calculated as waist (m) divided by height (m). Pulse pressure was reported as the difference between mean systolic and mean diastolic pressure. All participants were categorised as hypertensive or non-hypertensive at age 25 or 26, based on a definition by the American Heart Association (systolic blood pressure ≥130 mmHg or diastolic blood pressure ≥80 mmHg) [25].

### Outcomes

Nutritional status: BMI and WHtR; lipid levels: total cholesterol, HDL-cholesterol, LDL-cholesterol, and triglycerides; blood pressure: systolic, diastolic and pulse pressure; carotid intima-media thickness.

## Statistical analyses

Statistical tests were performed with SAS version 9.4 (SAS Institute, Inc, Cary, NC). Statistical significance was inferred at a 2-tailed P-value <.05.

Characteristics of study participants are reported as means with standard deviations (SDs) for continuous variables and as proportions for categorical variables. Differences between the two cohorts and between the STRIP intervention and control groups in birth weight and gestational age were analysed using t-tests, while for birth weight category, the Cochran-Mantel-Haenszel method was applied. The Cochran-Mantel-Haenszel method was also used for analyzing the association between birth weight category and elevated blood pressure at age 25 or 26. The main analyses examined the associations between birthweight category and longitudinal data on cardiovascular markers. For this purpose, a repeated measures ANOVA was used. Compound symmetry was used as covariance structure. In each model, one of the cardiovascular markers was included as the outcome variable along with birth weight category (SGA, AGA or LGA), sex and assessment time point (study wave in the ABC and assessment age in STRIP). Finally, an interaction term between assessment time point and birth weight category was included in the model to calculate least square means (adjusted means) with 95% confidence intervals for each assessment time point and to yield group-wise comparisons between the birth weight categories.

Additionally, to assess potential mediation, all models were adjusted for BMI. Linear regression analysis adjusted for sex was used to analyse effect of birth weight on cIMT and differences in cIMT between the birth weight categories.

## Results

Characteristics of the participants are reported in Table 1. Mean gestational age was lower in the ABC compared to the STRIP participants (38.9 ± 1.5 weeks vs. 39.4 ± 1.5 weeks, *p*<.0001). Mean birth weight was also lower in the ABC participants (3,043 ± 600 grams vs. 3,582 ± 501 grams, *p*<.0001). There were more SGA babies (22.4% vs 2.4%) and less LGA babies (8.3% vs. 29.2%) in the ABC than in the STRIP cohort (*p*<.0001).

**Table 1.** Characteristics of the study participants expressed as means with standard deviations or proportions (%). The number in parenthesis refers to the number of participants for the given variable.

	ABC	STRIP
	*mean ± SD (N)*	*mean ± SD*
**Birth**		
Gestational age, weeks	38.9 ± 1.5 (541)	39.4 ± 1.5 (747)
Birthweight, grams	3043 ± 600 (541)	3582 ± 501 (747)
SGA, %	22.4 (*N* = 121; 50% male)	2.4 (*N* = 18; 50% male)
AGA, %	69.3 (*N* = 375; 51% male)	68.4 (*N* = 511; 51% male)
LGA, %	8.3 (*N* = 45; 51% male)	29.2 (*N* = 218; 47% male)
**Age 11**		
Age, years	11.4 ± 1.2 (505)	11.0 ± 0.05 (592)
Weight, kg	35.4 ± 11.7 (504)	39.4 ± 8.3 (592)
Height, cm	143.4 ± 10.6 (504)	147.6 ± 6.8 (592)
BMI, kg/m²	16.9 ± 3.5 (504)	18.0 ± 2.9 (592)
WHtR	0.45 ± 0.05 (475)	0.43 ± 0.05 (577)
SBP, mmHg	107.7 ± 10.4 (492)	106.4 ± 10.3 (592)
DBP, mmHg	68.1 ± 7.1 (492)	58.6 ± 6.4 (592)
PP, mmHg	39.5 ± 7.6 (492)	47.8 ± 9.1 (592)
Total cholesterol, mmol/L	4.0 ± 0.8 (475)	4.5 ± 0.7 (586)
HDL, mmol/L	1.2 ± 0.3 (473)	1.3 ± 0.3 (585)
LDL, mmol/L	2.3 ± 0.7 (472)	2.8 ± 0.6 (585)
Triglycerides, mmol/L	1.2 ± 0.7 (475)	0.80 ± 0.4 (586)
**Age 18**		
Age, years	18.3 ± 1.1 (416)	18.0 ± 0.06 (487)
Weight, kg	60.1 ± 18.7 (415)	66.5 ± 12.2 (487)
Height, cm	167.1 ± 8.4 (415)	173.9 ± 9.0 (487)
BMI, kg/m²	21.4 ± 5.6 (414)	21.9 ± 3.4 (487)
WHtR	0.47 ± 0.08 (398)	0.44 ± 0.05 (485)
SBP, mmHg	109.8 ± 11.8 (403)	119.3 ± 13.3 (485)
DBP, mmHg	68.4 ± 7.8 (403)	62.5 ± 7.1 (485)
PP, mmHg	40.4 ± 6.9 (403)	56.8 ± 10.9 (485)
Total cholesterol, mmol/L	4.1 ± 0.9 (398)	4.2 ± 0.8 (481)
HDL, mmol/L	1.1 ± 0.3 (398)	1.3 ± 0.3 (482)
LDL, mmol/L	2.4 ± 0.7 (398)	2.5 ± 0.6 (481)
Triglycerides, mmol/L	1.4 ± 0.9 (398)	1.0 ± 0.4 (481)
**Age 25/26**		
Age, years	25.4 ± 1.2 (415)	26.1 ± 0.2 (526)
Weight, kg	66.7 ± 20.0 (411)	74.1 ± 16.0 (526)
Height, cm	167.3 ± 8.5 (414)	173.5 ± 9.3 (526)
BMI, kg/m²	23.8 ± 6.5 (411)	24.5 ± 4.4 (526)
WHtR	0.52 ± 0.1 (388)	0.47 ± 0.06 (526)
SBP, mmHg	110.9 ± 12.4 (396)	120.8 ± 10.9 (526)
DBP, mmHg	71.45 ± 8.6 (396)	71.9 ± 7.3 (526)
PP, mmHg	39.4 ± 6.7 (396)	48.8 ± 8.1 (526)
Total cholesterol, mmol/L	4.5 ± 1.1 (371)	4.6 ± 0.9 (524)
HDL, mmol/L	1.0 ± 0.3 (367)	1.3 ± 0.3 (524)
LDL, mmol/L	2.4 ± 0.8 (365)	2.8 ± 0.7 (524)
Triglycerides, mmol/L	1.5 ± 1.1 (369)	1.0 ± 0.5 (524)

BMI: Body Mass Index; WHtR: Waist-to-Height-Ratio; HDL: High-density Lipoprotein cholesterol; LDL: Low-density Lipoprotein cholesterol; SBP: Systolic Blood Pressure; DBP: Diastolic Blood Pressure; PP: Pulse Pressure; SGA: Small for Gestational Age; AGA: Appropriate for Gestational Age; LGA: Large for Gestational Age.

### Nutritional status

**Figure 1.**
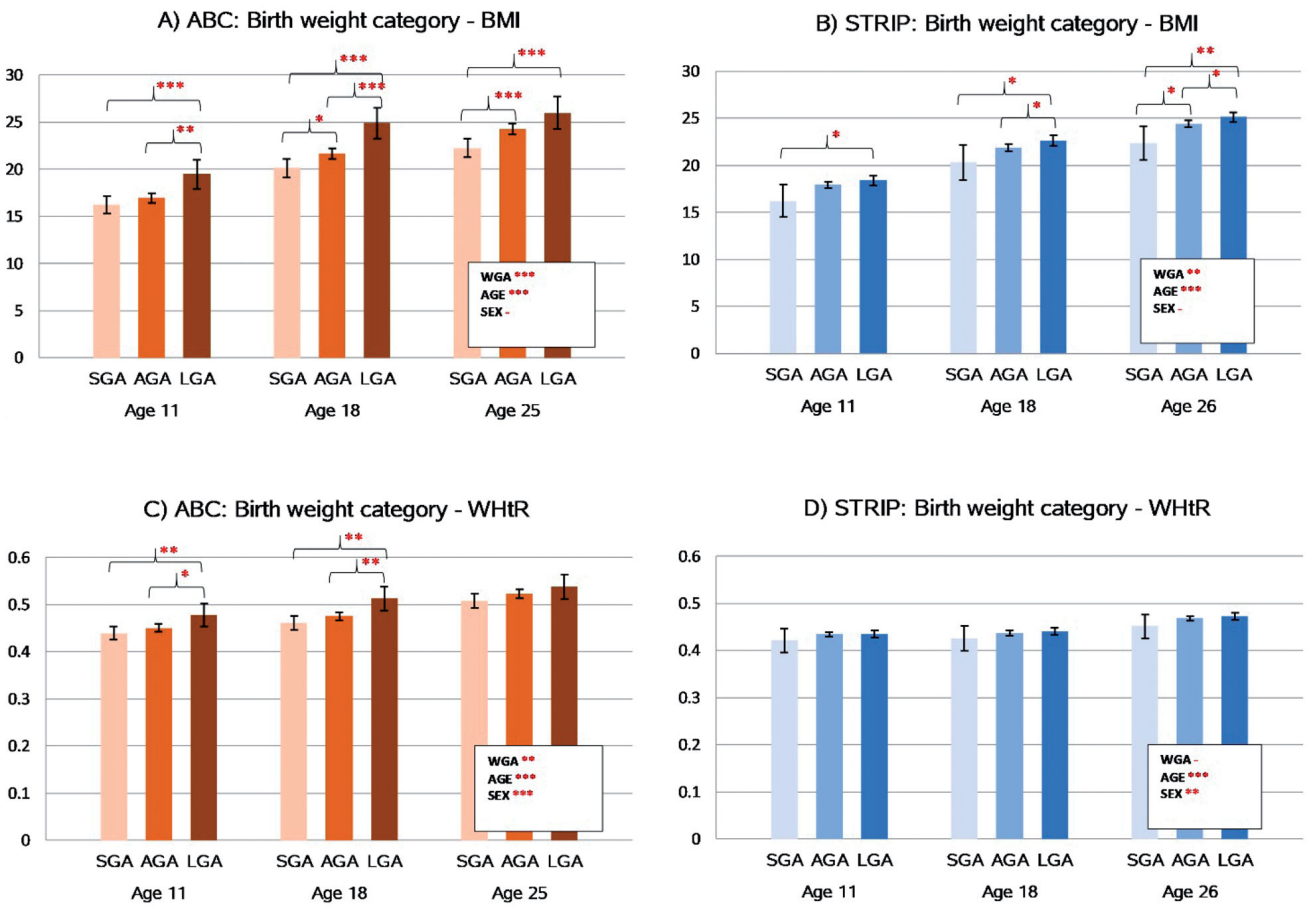
Associations between birthweight category and nutritional status in the ABC and STRIP cohorts. Bars indicate least adjusted means with error bars for 95% confidence intervals. The values in the boxes refer to the longitudinal analyses with WGA referring to birth weight category and AGE to assessment time point. Significant intracohortal differences between the birthweight categories at each follow-up are indicated with brackets. Unit for BMI is kg/m2. Abbreviations: BMI: Body Mass Index; WHtR: Waist-to-Height-Ratio; SGA: Small for Gestational Age; AGA: Appropriate for Gestational Age; LGA: Large for Gestational Age; WGA: Weight for Gestational Age category. Asterisks indicate statistical significance with *p<.05, **p<.01 and ***p<.001.

The associations between birth weight category and later nutritional status are presented in Figure 1. Birth weight category was associated with BMI in a step-wise manner from childhood to adulthood in both cohorts (*p*<.0001 for ABC and *p*=.003 for STRIP) with higher BMI found for higher birth weight category. Group-wise comparisons of the birth weight categories showed that participants who were LGA had higher BMI compared to the SGA participants in both the ABC and the STRIP study at all follow-ups. In the ABC, the sex and study point adjusted mean for BMI in the LGA group at age 11 was 19.5 kg/m^2^ (95%CI 17.9–21.0) vs. 16.2 kg/m^2^ (15.3–17.2, *p*=.0004) in the SGA group; at age 18 it was 24.9 kg/m^2^ (23.3–26.5) vs. 20.1 kg/m^2^ (19.1–21.1, *p*<.0001), and at age 25, 26.0 kg/m^2^ (24.3–27.7) vs. 22.3 kg/m^2^ (21.3–23.3, *p*=.0002). BMI was higher in the AGA group compared to the SGA group at age 18: 21.6 kg/m^2^ (21.1–22.2) vs. 20.1 kg/m^2^ (*p*=.01) and at age 25: 24.3 kg/m^2^ (23.8–24.9) vs. 22.3 kg/m^2^ (*p*=.0005). LGA participants had higher BMI than AGA participants at age 11 (19.5 kg/m^2^ vs. 17.0 kg/m^2^ [16.4–17.5, *p*=.0027] and at age 18 (24.9 kg/m^2^ vs. 21.6 kg/m^2^, *p*=.0002). In the STRIP study, BMI in the LGA group at age 11 was 18.4 kg/m^2^ (17.9–18.9) vs. 16.2 kg/m^2^ (14.5–18.0, *p*=.018) in the SGA group; at age 18, 22.7 kg/m^2^ (22.1–23.2) vs. 20.3 kg/m^2^ (18.4–22.1, *p*=.016) and at age 26, 25.1 kg/m^2^ (24.6–25.7) vs. 22.3 kg/m^2^ (20.5–24.1, *p*=.003). BMI was higher in the AGA group compared to the SGA group only at age 26: 24.4 kg/m^2^ (24.1–24.8) vs. 22.3 kg/m^2^ (*p*=.024). LGA participants had higher BMI than AGA participants at ages 18 (22.7 vs. 21.9 kg/m^2^ [21.6–22.3], *p*=.025) and at age 26 (25.1 vs 24.4 kg/m^2^, *p*=.022) (Figure 1).

Similar to BMI, birth weight category was associated in a step-wise manner with WHtR in the ABC (*p*=.004) with higher WHtR levels found for higher birth weight category. This association persisted after adjusting for BMI (*p*=.004). In contrast, there was no association between birth weight category and WHtR in the STRIP study (*p*=.33).

In the ABC, group-wise comparisons between the birth weight categories showed that WHtR was higher in the LGA participants compared to the SGA participants at ages 11 (0.48[0.45–0.50] vs. 0.44[0.45–0.45], *p*=.007) and 18 (0.51[0.49–0.54] vs. 0.46[0.44–0.48], *p*=.0007). Similarly, the LGA participants had higher WHtR than the AGA participants at ages 11 (0.48 vs. 0.45[0.44–0.46], *p*=.030) and 18 (0.51 vs. 0.48[0.47–0.48], *p*=.006). There were no differences in WHtR between the SGA and AGA participants at any studied age and no differences between any of the birth weight categories were found at age 25.

### Lipid levels

**Figure 2.**
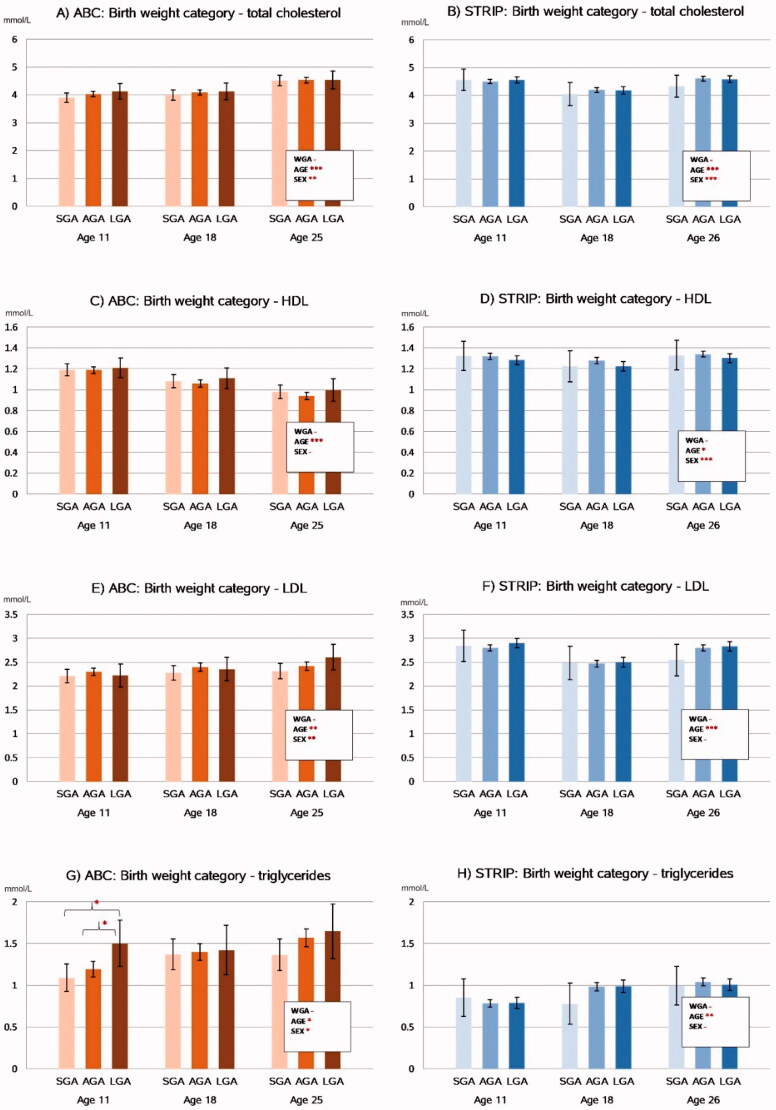
Associations between birthweight category and serum lipid levels in the ABC and STRIP cohorts. Bars indicate adjusted means with error bars for 95% confidence intervals. The values in the boxes refer to the longitudinal analyses with WGA referring to birth weight category and AGE to assessment time point. Significant intracohortal differences between the birthweight categories at each follow-up are indicated with brackets. Unit for total cholesterol, HDL, LDL and triglycerides is mmol/l. Abbreviations: HDL: High-density Lipoprotein; LDL: Low-density Lipoprotein; SGA: Small for Gestational Age; AGA: Appropriate for Gestational Age; LGA: Large for Gestational Age; WGA: Weight for Gestational Age category. Asterisks indicate statistical significance with *p<.05, **p<.01 and ***p<.001.

The associations between birth weight category and serum lipid levels are summarized in Figure 2. The longitudinal analyses revealed that there were no associations between birthweight category and total, HDL-, LDL-cholesterol, or triglyceride levels in the cohorts. Group-wise comparisons between the birth weight categories indicated that in the ABC at age 11, the LGA participants had higher triglyceride levels compared to SGA (1.50 mmol/l [1.22–1.78] vs. 1.09 mmol/l [0.93–1.26], *p* = 0.013) and AGA (1.50 vs. 1.20 mmol/l [1.10–1.29], *p* = 0.041) participants. These associations did not persist after adjusting for BMI.

### Blood pressure

**Figure 3.**
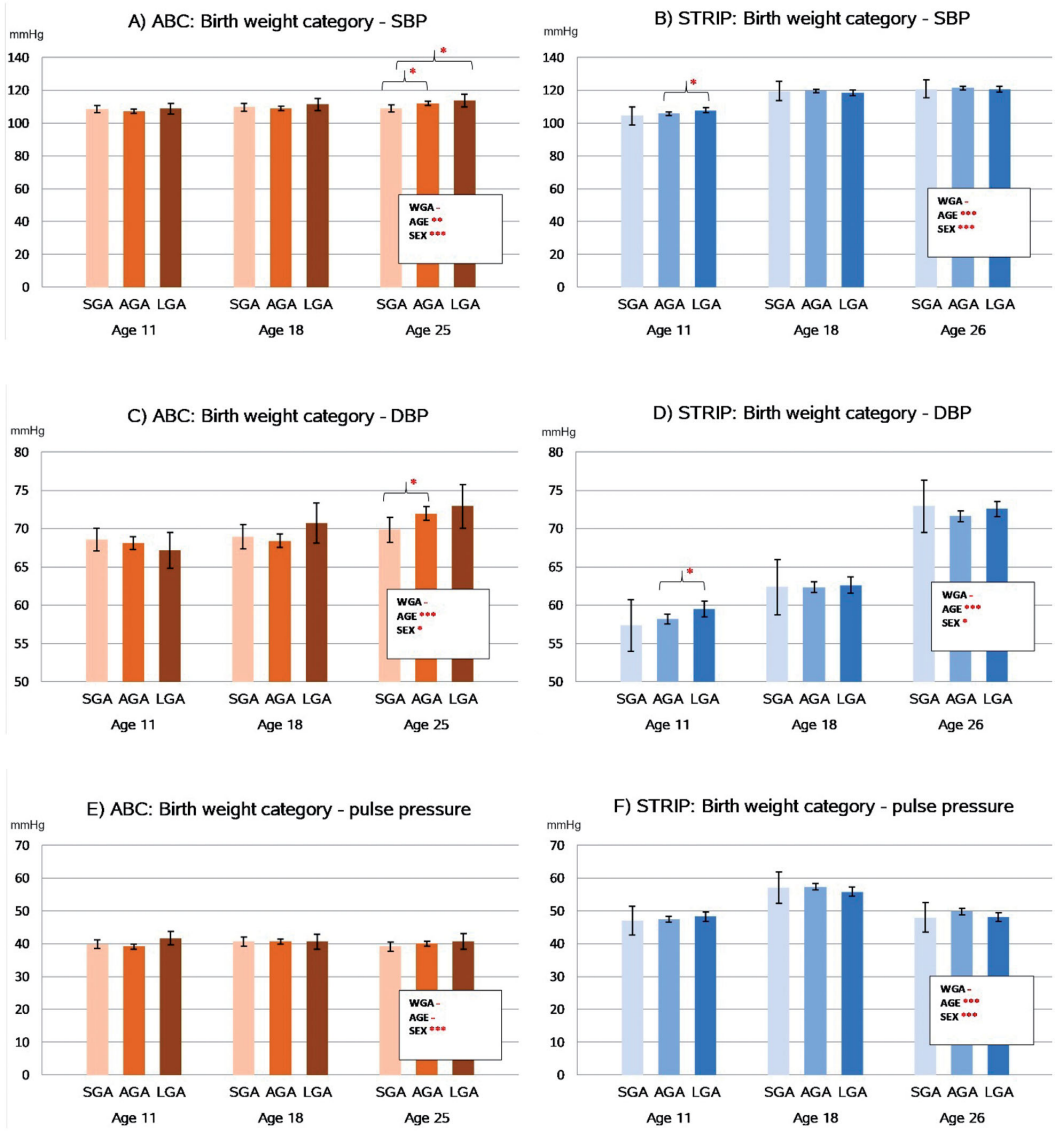
Associations between birthweight category and longitudinal trends in blood pressure levels in the ABC and STRIP cohorts. Bars indicate least square means with error bars for 95% confidence intervals. The values in the boxes refer to the longitudinal analyses with WGA referring to birth weight category and AGE to assessment time point. Significant intracohortal differences between the birthweight categories at each follow-up are indicated with brackets. Unit for SBP, DBP and pulse pressure is mmHg. Abbreviations: SBP: Systolic Blood Pressure; DBP: Diastolic Blood Pressure; SGA: Small for Gestational Age; AGA: Appropriate for Gestational Age; LGA: Large for Gestational Age; WGA: Weight for Gestational Age category. Asterisks indicate statistical significance with *p<.05, **p<.01 and ***p<.001.

The associations between birth weight category and future blood pressure levels are presented in Figure 3.

The longitudinal analyses revealed that birth weight category was not associated with systolic blood pressure levels in either cohort. Group-wise comparisons of the birth weight categories, however, showed that in the ABC cohort at age 11, systolic blood pressure was higher in the LGA group compared to the SGA group (113.7 mmHg [109.7–117.7] vs. 109.0 mmHg [106.7–111.3], *p*=.046), and in the AGA group compared to the SGA group (112.0 mmHg [110.7–113.2] vs. 109.0 mmHg, *p*=.028). Systolic blood pressure was higher in the LGA group compared to the AGA group (113.7 mmHg vs. 112.0 mmHg, *p*=.037). These associations did not persist after adjusting for BMI.

In the longitudinal analyses, birth weight category was also not associated with diastolic blood pressure levels in the ABC or STRIP cohorts. In the group-wise analyses, AGA participants had higher diastolic blood pressure than SGA participants in the ABC at age 25 (71.9 mmHg [71.1–72.8] vs. 69.9 mmHg [68.2–71.5] *p*=.027). In the STRIP study, LGA participants had higher diastolic blood pressure than AGA participants at age 11 (59.5 mmHg [58.5–60.5] vs. 58.2 mmHg [57.5–58.8], *p*=.031) (Figure 3). These associations did not persist after adjusting for BMI. There were no associations between birth weight category and pulse pressure in either cohort.

In the STRIP study, 17.0% (*N* = 127) and in the ABC 12.9% (*N* = 70) of participants had elevated blood pressure at age 25 or 26. There was no association between birth weight category and elevated blood pressure in either cohort (*p*=.34 for STRIP, *p*=.36 for ABC).

### Carotid intima-media thickness

**Figure 4.**
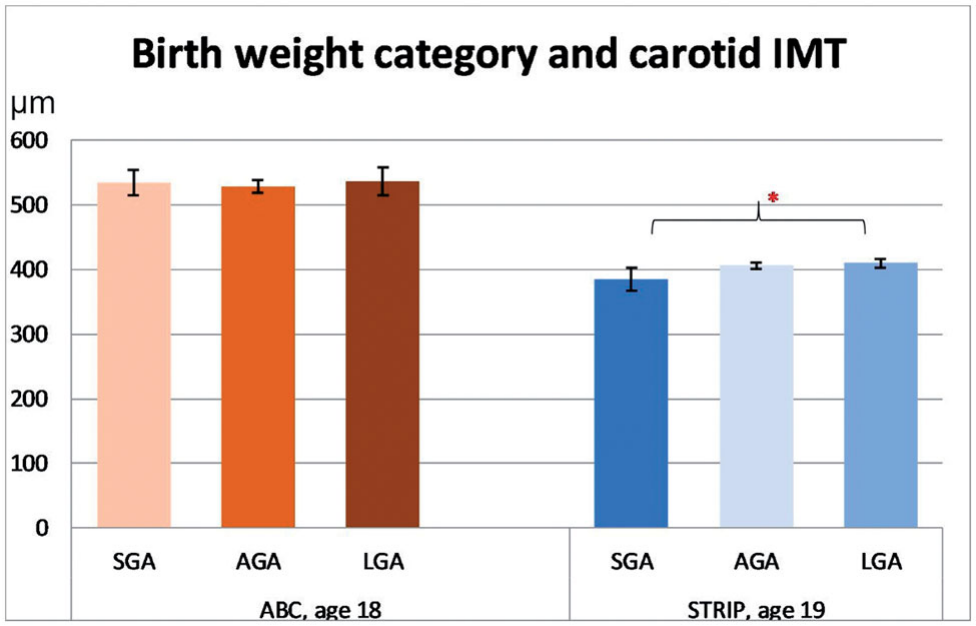
Carotid intima media thickness (IMT) according to birth weight category in the cohorts. Significant intracohortal differences between the birthweight categories at each follow-up are indicated with brackets. Asterisks indicate statistical significance with *p<.05, **p<.01 and ***p<.001. Values are in µm.

Mean values for cIMT with 95% confidence intervals at age 18/19 years for each birth weight category are presented in Figure 4. In the ABC, there was no association between birth weight category and cIMT (*p* = 0.72). Similarly, group-wise comparisons revealed no differences in cIMT between the birth weight categories.

In the STRIP study, there was no association between birth weight category and cIMT (*p*=.19). However, in the group-wise comparisons, SGA participants had a tendency for lower cIMT than AGA participants (386 µm [367–406] vs. 407 µm [402–412], *p*=.085) and had lower cIMT than LGA participants (386 µm vs. 409 µm [403–416], *p*=.024).

## Discussion

The present study shows that birth weight for gestational age is associated with later cardiovascular health in these two unique, distinct cohorts from different parts of the world. The strongest associations were found between birth weight category and BMI, with SGA infants having lower BMI and LGA infants higher BMI throughout the follow-ups in both cohorts. Interestingly, WHtR, an indicator of central adiposity, was associated with birth weight category in the Indigenous Australian ABC cohort, but not in the Finnish STRIP study. Future lipid levels showed to be largely independent of birth weight category in both cohorts. However, in the ABC at age 11, LGA participants had higher triglyceride levels than SGA and AGA participants. Birth weight category was also associated with blood pressure levels in both cohorts. In the STRIP cohort, LGA participants had higher systolic and diastolic blood pressure than AGA participants at age 11. In the ABC, differences in blood pressure were only seen at age 25 when SGA participants had lower systolic blood pressure than AGA and LGA participants and lower diastolic blood pressure than AGA participants. When adjusting for BMI, the associations found for blood pressure and lipid levels did not persist, indicating that BMI likely mediates these associations. Birth weight category was associated with cIMT only in the STRIP cohort, where SGA participants had lower cIMT than LGA participants at age 19.

The observed association between birth weight and BMI from childhood to adulthood is in line with prior studies showing that high birth weight may lead to obesity later in life [26–28]. In a cross-sectional study on Korean adolescents (*N* = 1304), higher birth weight was associated with both higher BMI and higher fat mass index [26] and a study on 6 to 11 year old Canadians showed that every 100 g increase in birth weight was associated with a 5% increase in a child’s obesity risk [28]. Moreover, there is growing evidence that obesity and the association between birth weight and future BMI could at least in part be explained by polygenic inherited susceptibility to obesity, suggesting a shared background for the phenotypes. As an example, a genome-wide polygenic score to quantify the genetic risk for future obesity was generated in a genome-wide association study and it was found that this genetic risk score was associated with only small differences in birth weight but that these differences grew in size with time reaching a gradient of 12 kg by the age of 18 between top and bottom risk score deciles [34].

Collectively, genetic and epigenetic properties are thought play an important role in the association between birth weight and later CVD risk. The association between low birth weight and later CVD has been documented in numerous epidemiological studies [29–31] Foetal epigenetic programming in a suboptimal intrauterine environment is suggested to drive the processes leading to greater cardiovascular risk in adulthood [32]. More recently, it has been proposed that foetal overnutrition leading to high birth weight also causes epigenetic programming that increases the individual’s risk of CVD in adulthood [33].

A large multi-cohort study from Finland and the UK examined the role of birth weight adjusted for gestational age on later metabolic profile and found that lower birth weight was adversely associated with cardiometabolic biomarkers such as lipid levels and inflammatory markers in adulthood. The magnitude of this association, however, was modest, similar to that caused by high BMI in adulthood. It was suggested similar molecular pathways may underlie both low birth weight and adulthood overweight [35].

Somewhat unexpectedly, only few associations between birth weight category and blood pressure or lipid levels were found in the present study, whereas previous studies have reported inverse associations between these variables. In the Bogalusa Heart Study (Louisiana), lower birth weight was associated with both higher blood pressure levels (systolic, diastolic and pulse pressure) [36] from childhood to adulthood as well as higher LDL cholesterol and triglyceride levels in adolescence [37]. A large (*N* = 300.000), genome-wide association study using data from the EGG consortium and the UK biobank found evidence to support that the inverse association between birth weight and blood pressure is attributable both directly to the foetal genotype and indirectly to maternal genetic factors that produce an adverse intrauterine environment. According to the study, it is possible that some of the same alleles that are associated with lower birth weight might also cause higher blood pressure later in life [38]. The lack of association between birth weight and blood pressure and lipid levels in our study may be due to the relatively small sample sizes.

The finding that birth weight was associated with WHtR in the ABC but not in the STRIP cohort, may be explained by different patterns of fat accumulation in the populations. In a previous study examining the prevalence of metabolic syndrome in the ABC, it was noted that large waist circumference was quite common despite low rates of overweight and the relative underweight nature of the cohort. It was suggested that there could be a susceptibility for central fat accumulation in the population and that central adiposity could serve as a better predictor of metabolic and cardiovascular disorders than BMI also in Indigenous Australians [39]. Another study compared body fat distribution between adults of Aboriginal and European ancestry in Australia and concluded that there were significant differences in body shape with Aboriginal women having larger waist circumferences than their European Australian counterparts [40]. A study from the ABC confirmed that especially females in the Aboriginal population are more likely to be affected by central obesity [41].

A key finding of this study was that larger babies, although born in completely different global contexts, tend to have larger BMIs already in childhood, putting these individuals at greater risk for obesity-related disorders such as type 2 diabetes and cancer [42,43]. A previous study examining tracking of obesity in the ABC found a tendency for overweight children to remain overweight as adults [41], a phenomenon seen in many other populations as well [44]. With the proportion of newborns with high birth weight increasing in many populations [45–47], and the obesity pandemic posing a major global health threat [48], the effect of birth weight on future BMI is of evident importance. The rising rates of high birth weight infants have been explained by maternal factors such as pre-pregnancy obesity, gestational weight gain, and gestational diabetes [49], indicating a need to focus on maternal health to prevent childhood obesity and help tackle the obesity pandemic [50]. On the other hand, there is evidence that if childhood obesity discontinues into adulthood, the risk for later type 2 diabetes, hypertension, dyslipidemia, and atherosclerosis is similar to those who were never obese [51], implicating the benefits of interventions in childhood or even earlier [52].

A particular strength of this study is that we were able to compare two unique cohorts that were established almost simultaneously on opposite sides of the globe in very different socioeconomic and cultural settings. Both of these longitudinal studies began in infancy, and have had good retention rates and systematically structured follow-ups. We acknowledge that comparisons between the cohorts remain observational, as the methodologies to assess anthropometrics and blood pressure, blood sampling and carotid ultrasonography were not standardised across the cohorts to allow for statistical comparisons. As the sample sizes of both studies and especially the number of SGA infants in the STRIP cohort and the number of LGA infants in the ABC were quite low, it is possible that some existing associations were not found. Parental effects on birth weight were not analysed although especially maternal risk factors such as gestational diabetes and maternal BMI could add important information about the intergenerational inheritance of cardiovascular risk. Finally, clinical relevance of the cardiovascular risks described in this paper remain to be analysed in later studies, as the participants were still young with little cardiovascular morbidities.

Collectively, findings of this study suggest that birth weight category is associated with later cardiovascular risk profile with the most robust associations seen in nutritional status indicated by BMI and WHtR. This finding supports targeted prevention strategies for those individuals at risk to improve cardiovascular health worldwide. Additional research on the longitudinal health trajectories of small and large birth weight infants is needed for evaluation of the extent of cardiovascular risk related to birth weight. Future research endeavours may focus on the possibilities of incorporating birth weight into CVD risk assessment and on the impact of targeted intervention strategies for individuals at risk based on their birth weight.

## Supplementary Material

Supplemental Material

## Data Availability

Data may be obtained from a third party and are not publicly available. All data are stored confidentially and are not freely available in the public domain, but specific proposals for collaboration are welcomed. Collaborations are established through formal agreement with the ABC steering committee. Contact information: abcstudy@menzies.edu.au.
