# Supplementary material for: Birth weight for gestational age and later cardiovascular health: a comparison between longitudinal Finnish and indigenous Australian cohorts
Source: Ann Med. 2021 Nov 10;53(1):2060–71. doi: 10.1080/07853890.2021.1999491 (PMC8583840; doi:10.1080/07853890.2021.1999491)
Supplement: Supplemental Material [file IANN_A_1999491_SM4651.docx]

Supplementary data


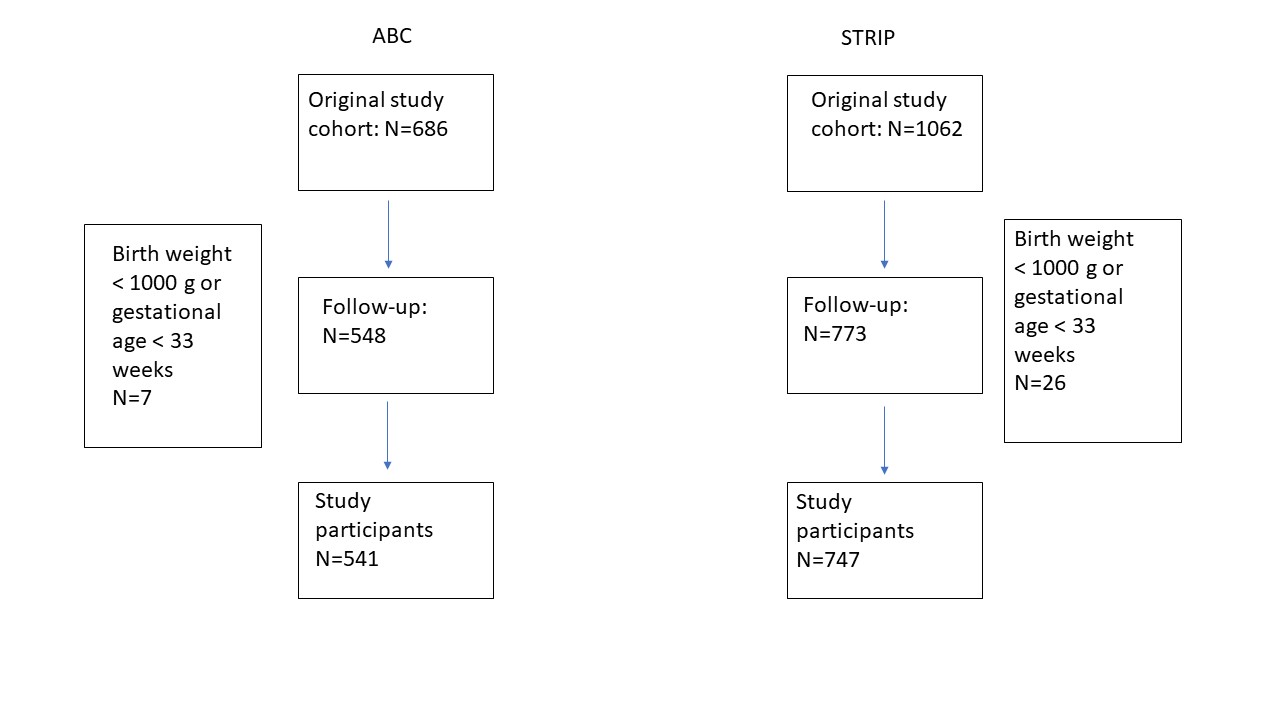


Supplementary figure 1. Flow chart depicting participant selection in the two cohorts
